# Unraveling the Role of STK11/LKB1 in Non-small Cell Lung Cancer

**DOI:** 10.7759/cureus.21078

**Published:** 2022-01-10

**Authors:** Vikram Sumbly, Ian Landry

**Affiliations:** 1 Internal Medicine, Icahn School of Medicine at Mount Sinai, New York City Health and Hospitals/Queens, Jamaica, USA

**Keywords:** lkb1/ampk/mtor, pathophysiology, stk11/lkb1, lung cancer, oncology

## Abstract

There are two major groups of lung cancer: non-small cell lung cancer (NSCLC) and small cell lung cancer (SCLC). NSCLCs can be further separated into three different categories: lung adenocarcinoma, squamous cell carcinoma, and large cell carcinoma. Pulmonary adenocarcinomas represent nearly half of all lung cancer cases and are known to be caused by smoking, certain occupational exposures, and specific genetic mutations. Scientists have noticed that most NSCLCs are driven by defects in the following genes: EGFR, BRAF, ALK, MET, and HER. Abnormalities in the STK11/LKB1 gene have also been shown to induce lung adenocarcinoma. LKB1-deficient cancer cells contain an overactive AMPK “energy sensor,” which inhibits cellular death and promotes glucose, lipid, and protein synthesis via the mTOR protein complex. Studies have also discovered that the loss of STK11/LKB1 favors oncogenesis by creating an immunosuppressive environment for tumors to grow and accelerate events such as angiogenesis, epithelial-mesenchymal transition (EMT), and cell polarity destabilization. STK11/LKB1-mutant lung cancers are currently treated with radiotherapy with or without chemotherapy. Recent clinical trials studying the effects of glutaminase inhibitors, mTOR inhibitors, and anti-PD-L1 therapy in lung cancer patients have yielded promising results. This narrative review provides an overview of the STK11/LKB1 gene and its role in cancer development. Additionally, a summary of the LKB1/APMK/mTOR is provided.

## Introduction and background

Non-small cell lung cancers (NSCLCs) represent approximately 85% of all lung cancer cases and can be subdivided into three major groups: adenocarcinoma, squamous cell carcinoma, and large cell carcinoma [1]. Lung adenocarcinoma is the most common histological subtype as it accounts for 40%-50% of all NSCLCs [2,3]. Lung cancer remains the leading cause of cancer-related death in the United States of America. Although most lung cancer cases are linked to cigarette smoking, epidemiologic studies suggest that approximately 10% of all lung cancer patients have no smoking history [2]. There are other risk factors associated with the development of lung cancer, which include a poor diet, occupational exposures, and genetic predispositions [4,5]. According to Global Cancer Incidence, Mortality And Prevalence (GLOBOCAN), the incidence and mortality rate of lung cancer are two times higher in men than in women [6]. Most patients have a poor prognosis with a median five-year overall survival of roughly 10%-20% [6].

It is well established that certain mutations in critical genes initiate tumorigenesis and confer an invasive phenotype to lung cancer cells. Although a significant portion (~50%) of NSCLCs are linked to mutations in KRAS and EGFR, other implicated genes include BRAF (5%), ALK (3%), MET (3%), and HER2 (3%) [7]. STK11/LKB1 was first discovered and recognized as an important tumor suppressor gene in the 1990s after analyzing the genomic sequence of patients with Peutz-Jeghers syndrome [8]. STK11/LKB1 mutations cause abnormal mTOR and HIF-1‐α expression patterns due to the partial loss of AMPK regulation [8]. Della Corte et al. have observed that lung adenocarcinomas routinely harbor STK11/LKB1 mutations and that 7% of these mutations co-occur with a KRAS mutation [9]. Patients with STK11/LKB1 mutations are often resistant to immunotherapy and have a worse prognosis than their STK11/LKB1 negative counterparts [10].

## Review

A brief overview of the SKT11/AMPK axis

**Figure 1:**
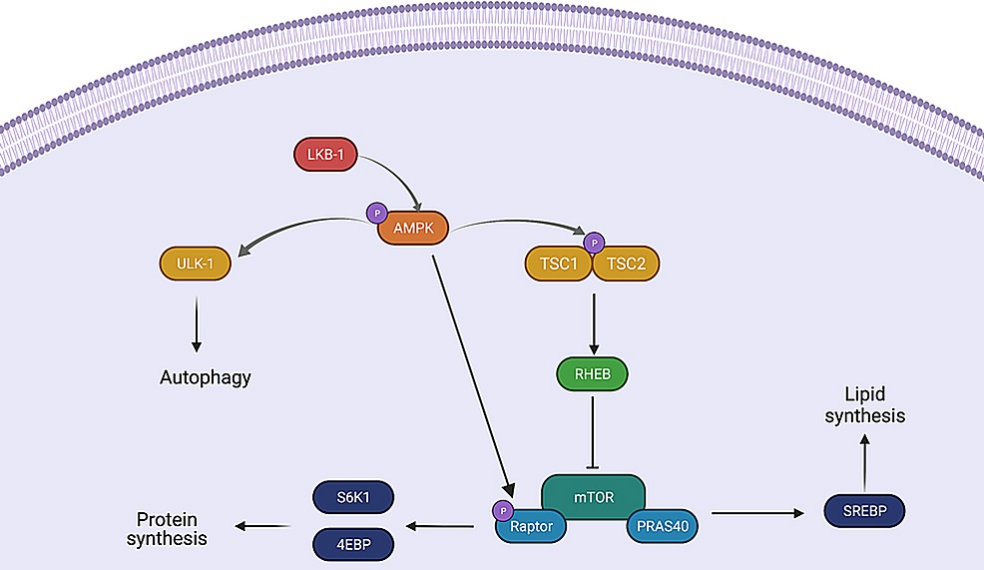
LKB1/mTOR/AMPK biochemical cascade

LKB1 is a master kinase that serves as an “energy sensor” and plays a critical role in the conservation of ATP. This enzyme phosphorylates AMP-activated protein kinase (AMPK), which in turn promotes the production of ATP and inhibits the ATP-consuming processes [11]. In nutrient-scarce conditions, AMPK inhibits cell growth via the suppression of the mammalian target of the rapamycin (mTOR) pathway (Figure 1) [12]. AMPK rapidly suppresses mTORC1 by activating tuberous sclerosis 1- tuberous sclerosis 2 (TSC1-TSC2) tumor suppressor complex and the mTORC1 binding subunit raptor [13]. Phosphorylation of the TSC1-TSC2 complex leads to the inhibition of Ras homolog enriched in the brain (RHEB), an activator of the mTORC1 complex, while the phosphorylation of raptor directly inhibits the mTORC1 complex [12]. In humans, this mTOR biochemical cascade is driven by the mTORC1 and mTORC2 protein complexes. mTORC1 is composed of mTOR, proline-rich Akt substrate of 40 kDa (PRAS40), mammalian lethal with SEC13 protein 8 (mLST8), and raptor (regulatory associated protein of mTOR). mTORC2 is composed of mTOR, mLST8, mammalian stress-activated protein kinase-interacting protein 1 (mSIN1) and rictor (rapamycin-insensitive companion of mTOR) [14]. Increased mTORC1 signaling allows for protein and lipid biosynthesis, which are important for cell growth, proliferation, and migration [15]. The phosphorylation of two effectors, p70S6 Kinase 1 (S6K1) and EIF4E binding protein (4EBP), by mTORC1, is important for the synthesis of protein [15]. Indeed, these proteins are necessary for efficient 5’ cap-dependent messenger ribonucleic acid (mRNA) translation to occur [16]. mTORC1 also uses sterol responsive element binding protein (SREBP) transcription factors for lipogenesis [17]. SREBP-dependent lipogenesis involves the activation of various factors such as CREB regulated transcription coactivator 2 (CRTC2), ribosomal protein S6 kinase beta-1 (S6K1), and lipin-1 [18].

Although the intracellular process of autophagy is normally inhibited by mTORC1, it can be activated by AMPK to further preserve cellular energy [19]. AMPK controls this phenomenon through the activation of the Unc-51 like autophagy activating kinase 1 (ULK-1) [20,21]. With the help of the autophagy-related protein 13 (ATG13) and scaffold protein FIP200, ULK-1 triggers the initial steps of autophagy which are then fine-tuned by proteins such as beclin1 (BECN1), autophagy-related protein 101 (ATG101), and vacuolar sorting 34 (VPS34) [22].

STK11/LKB1 deficiency and cancer

A wide variety of tumors have shown STK11/LKB1 aberrancies [23-25]. Genetic anomalies such as loss of heterozygosity, insertion-deletion (indel) mutations, and chromosomal deletions are well-known causes of loss of function in genes [26]. Genetic mapping revealed that STK11 is located on chromosome subband 19p13.3 [27]. This gene encodes for liver kinase B1 (LKB1), a protein kinase involved in cellular energy homeostasis [28]. In cancer cells with oncogenic driver mutations (e.g. KRAS, EGFR, or ALK), LKB1 deficiency has been observed to accelerate tumor development by inducing metabolic reprogramming of glucose, lipid, glutamine, and serine [29].

Shaw et al. noticed that LKB1 knockout mice were prone to hyperglycemia [30]. Hyperglycemic environments support tumor development by contributing to cancer cell proliferation, antiapoptosis, and invasiveness [29,30]. Tumor cells overexpress glucose transporters to ensure a continuous source of glucose for various metabolic pathways that will favor tumor growth at the expense of normal cells [31]. This process is expedited by the activation of other proteins such as epidermal growth factor (EGF), EGF receptor (EGFR), and peroxisome proliferator-activated receptor alpha (PPAR- α) [32].

In normal cells, the conversion of acetyl-CoA into malonyl-CoA by acetyl-coenzyme A carboxylase (ACC) is an important biochemical event that facilitates fatty acid synthesis and inhibits fatty acid oxidation within the mitochondria [33]. Sterol regulatory element-binding protein 1 (SREBP1) also tightly controls fatty acid and cholesterol synthesis by regulating the expression of different proteins such as ACC, fatty acid synthase (FASN), and ATP citrate lyase (ACYL) [33,34]. Both ACC and SREBP1 are normally inhibited by AMPK, but in LKB-1 deficient cells, these enzymes are continuously active and permit the accumulation of lipid [29]. Tumor cells require high levels of fatty acids for membrane synthesis and cell proliferation [33].

Implications of SKT11 mutations in NSCLC

STK11/LKB1 mutations are more prevalent in lung adenocarcinomas but are also found in pulmonary squamous cell carcinoma and large cell adenocarcinoma [35]. These mutations routinely co-occur with KRAS mutations and seem to be associated with a decreased overall survival due to their ability to create aggressive tumors with metastatic features [35,36]. Shen et al. discovered that STK11/LKB1 mutations have a slightly higher predilection for Caucasian patients (16%) than Asian patients (4%-7%) [37]. African Americans were also prone to these mutations, which are primarily believed to be large intragenic, nonsense, and frameshift mutations [38,39]. Some mutational hotspots include Q37X, 837-842delC, and frameshift indels at codons 51-53 [39].

Sanchez-Cespedes et al. reported that LKB-1 mutant lung cancer cells improperly activated AMPK or inhibited mTOR [39]. The loss of STK11/LKB1 seems to facilitate tumorigenesis and metastasis by increasing the expression of pro-angiogenic genes such as Nedd9, Vegfc, Loxl1, PDGF receptor, and Mmp2 [40]. The loss of LKB1 has also been observed to increase the production of S-adenosylmethionine (SAM), an important cofactor involved in deoxyribonucleic acid (DNA), RNA, and histone methylation [41]. This leads to the downregulation of stimulator of interferon genes (STING) and consequentially program death ligand 1 (PD-L1) [11]. Tumor cells with an abnormal STK11/LKB1 signaling axis were more likely to be surrounded by a “cold” immunosuppressive microenvironment characterized by the lack of inflammatory immune cells and the presence of multiple immunosuppressive cells (T-regulatory cells and tumor-associated neutrophils) [11,42].

LKB1 works together with MAP/microtubule affinity-regulating kinases (MARK) to maintain proper cellular polarity [43]. Lung cancer cells with STK11/LKB1 displayed various aberrancies such as improper Golgi positioning, lamellipodia formation, and morphology [44]. Furthermore, it is hypothesized that the lack of LKB1 also promotes epithelial-mesenchymal transition (EMT), a complex phenomenon that forces differentiated cells to re-acquire stem-cell-like properties [45]. Indeed, a study revealed that immortalized lung epithelial cells with LKB1 knockdown had increased invasiveness due to the heightened expression of zinc finger E-box binding homeobox 1 (ZEB1), a well-known EMT inducer [46].

Recent discoveries and future directions

Locally advanced NSCLC is often treated with radiotherapy with or without chemotherapy. A recent study by Sitthideatphaiboon et al. investigated the association between STK11 mutations in stage III NSCLC and outcomes after radiotherapy in a population of 194 patients [47]. After a median follow-up of four years, the study found that STK11 mutations were associated with a significantly higher locoregional recurrence rate (LRR, p=0.0108) and shorter disease-free survival (DFS; HR 2.53; [1.37-4.65], p=0.002). Additionally, five-year DFS and overall survival (OS) were significantly reduced in the STK11 group (DFS: 0% vs 14.6%, p=0.002; OS: 15.6% vs 42%, p=0.0228). LRR was also significantly higher in the STK11 group after concurrent chemoradiation (27.3% vs 9.0%, p=0.0023). These findings were consistent with the hypothesis of LKB1 loss influencing resistance to radiotherapy via the activation of the KEAP1/NRF2 pathway. LKB1 loss increases downstream production of NRF2, which, in turn, reduces reactive oxygen species (ROS) levels. Because radiation treatment relies on the destruction of tumor cells via intracellular ROS damage, tumor cells with these mutations can resist high levels of treatment [47].

The exploitation of energetic reactions to cellular stress has been shown to be a significant vulnerability of LKB1-deficient tumors [48]. In the presence of LKB1, normal cells will activate AMPK causing restricted growth and prolonged survival. However, tumors which lack LKB1 are very sensitive to apoptosis as they have an inability to restore their ATP levels [48]. These findings led to several retrospective analyses suggesting a correlation between oral biguanide, metformin, and reduced cancer risk [49-52]. While metabolic adaption to targeted therapies often occurs, the combination of LKB1-targeted treatments in conjunction with other therapies has the potential to increase the overall response of treatment.

The results of the CheckMate-059 trial showed that patients with advanced NSCLC, which progressed on platinum-based therapies achieved a significantly higher overall survival when treated with nivolumab, an anti-PD-L1 therapy (1 yr OS: 51% [Nivolumab] vs 39% [Docetaxel], with median survival 12.2 months vs 9.4 months [p=0.002]) [53]. Additionally, patients exhibited greater efficacy and had significantly fewer adverse effects (10% vs 54%) on nivolumab. Mutations in STK11 confer resistance to PD-L1 therapy and are often associated with co-mutations in KRAS, making the selection of treatment for STK11 mutated NSCLC a significant challenge [54,55].

Several studies are currently undergoing or recruiting to evaluate the response, safety, and outcomes in patients with advanced NSCLC and STK11 mutations (Table 1). Most of the current clinical trials are investigating the safety and efficacy of glutaminase inhibitors (BeGIN trial, KEAPSAKE, NCT04471415), while others are experimenting with the use of PD-L1 inhibitors in conjunction with glutaminase inhibitors or mammalian target of rapamycin (mTOR) inhibitors (BUNCH) [56]. Devarakonda et al. recently reported the results of their phase II study which evaluated everolimus (a dual inhibitor of mTOR) as a possible treatment for patients with solid malignancies harboring several mutations, including STK11 [56]. Of the eight patients available for analysis, one patient had a complete response (12.5%), one patient had stable disease (12.5%), and six of the patients (75%) progressed while on therapy. While everolimus was well tolerated, it was noted that grade 4 pericardial effusion was present in one patient. Interestingly, the patients with complete response or stable disease both harbored NF1 or STK11 mutations. These findings were suggestive of possible use for everolimus in this subset of patients.

**Table 1:** Recent and current clinical trials evaluating STK11 Abbreviations: NSCLC, non-small cell lung cancer; STK11, serine/threonine kinase 11; RR, response rate; TSC1, tuberous sclerosis complex 1

Study	Disease	Intervention	Mechanism of Action	Status
LUNG-MAP trial NCT04173507 [57]	Recurrent or metastatic NSCLCs with STK11 gene mutation	Talazoparib & Avelumab	PARP inhibitor & PD-L1 inhibitor	Recruiting
BUNCH trial NCT04518137 [58]	Advanced solid tumors harboring NFE2L2, STK11, RICTOR mutations	ATG-008 (onatasertib)	Dual inhibitor of mammalian target of rapamycin (mTOR)	Recruiting
NCT03611868 [59]	Metastatic melanoma or advanced solid tumors (i.e. NSCLC)	APG-115 & Pembrolizumab	Small-molecule mouse double minute 2 homolog (MDM2) inhibitor & PD-L1 inhibitor	Phase 1 complete
BeGIN trial NCT03872427 [60]	Metastatic or unresectable solid neoplasms with NF1, KEAP1/NRF2, or STK11	Telaglenastat hydrochloride	Glutaminase inhibitor	Recruiting
KEAPSAKE trial NCT04265534 [61]	Metastatic NSCLC with KEAP1/NRF2-mutations and STK11 mutation	Telaglenastat & Pembrolizumab & Carboplatin/Pemetrexed & Placebo + Pembrolizumab + Carboplatin/Pemetrexed	Glutaminase inhibitor & PD-L1 inhibitor & Alkylating agent / Antimetabolite	Active, not yet recruiting
KRYSTAL-1 trial NCT03785249 [62]	Advanced solid tumor malignancies with KRAS G12C mutation stratified by co-mutation status (e.g., STK11)	MRTX849 (Adagrasib)	KRAS inhibitor	Recruiting
NCT04471415 [63]	Advanced solid tumors, NSCLC	DRP-104 (Sirpiglenastat) & Atezolizumab	Glutaminase inhibitor & PD-L1 inhibitor	Recruiting
NCT04933695 [64]	Metastatic NSCLC with KRAS mutation and either PD-L1 or STK11 mutations	Sotorasib	KRAS G12C inhibitor	Not yet recruiting
NCT02352844 [65]	Solid malignancies with somatic inactivating mutations in tuberous sclerosis complex 1 (TSC1) gene	Everolimus	Dual inhibitor of mammalian target of rapamycin (mTOR)	Completed
CheckMate-057 NCT01673867 [66]	NSCLC tumors which progressed during or after platinum-based doublet therapy	Nivolumab & Docetaxel	PD-L1 inhibitor & Taxoid - microtubule assembly	Overall Survival OS: 12 m (N) vs 9.4 m (D); HR 0.73 (0.59-0.89), p=0.002 1y OS: 51% (N) vs 39% (D)

## Conclusions

This literature review depicts the association of STK11/LKB1 gene mutations and lung adenocarcinoma. Aberrancies in the STK11/LKB1 gene facilitate carcinogenesis by accelerating glucose, lipid, and protein biosynthesis through the LKB1/AMPK/mTOR biochemical axis. Such severe metabolic shifts involve the upregulation of glucose transporters and continuous activation of ACC and SREBP1. Most lung cancer cells harboring STK11/LKB1 mutations also lose their polarity, acquire stem cell-like properties, and resist the host’s immune system. Although STK11/LKB1 mutant lung cancers carry an unfavorable prognosis, the administration of different chemotherapeutic agents that target mTOR, glutaminase, and PD-L1 has shown to increase survival in NSCLC patients. However, more clinical trials with longer follow-up times are required to better understand the long-term benefits of targeted therapies in patients with STK11/LKB1 mutant lung cancer.
